# Prevalence of Endo-Periodontal Lesions in a Teaching Hospital of the University of Buenos Aires: A Cross-Sectional Study

**DOI:** 10.3390/dj14060347

**Published:** 2026-06-05

**Authors:** Stefania H. Caceres, Facundo Caride, Johana Castelllanos, Juliana Bugiolachi, Constanza Pontarolo, Nagore Ambrosio, Elena Figuero, Pablo A. Rodriguez

**Affiliations:** 1 Faculty of Dentistry, Complutense University of Madrid, 28040 Madrid, Spain; 2 Periodontics Department, Faculty of Dentistry, University of Buenos Aires, Buenos Aires C1122, Argentina; facundo.caride@odontologia.uba.ar (F.C.); johana.castellanos236@gmail.com (J.C.); juliana.bugiolachi@odontologia.uba.ar (J.B.); constanza.pontarolo@odontologia.uba.ar (C.P.); 3 ETEP (Etiology and Therapy of Periodontal and Peri-Implant Diseases) Research Group, Faculty of Dentistry, Complutense University of Madrid, 28040 Madrid, Spain; nambrosio@ucm.es (N.A.); elfiguer@ucm.es (E.F.); 4 Endodontics Department, Faculty of Dentistry, University of Buenos Aires, Buenos Aires C1122, Argentina; prodriguez@odontologia.uba.ar

**Keywords:** endo-periodontal lesions, periodontitis, prevalence, diabetes

## Abstract

**Background/Objectives**: In 2018, a classification system for periodontal and peri-implant diseases and conditions defined an endo-periodontal lesion (EPL) as a pathological communication between the endodontic and periodontal tissues of a given tooth. As the evidence to define the etiology, diagnosis, prognosis and treatment was considered limited, a cross-sectional study was carried out to evaluate its prevalence in a population treated at the Periodontics Department of Faculty of Dentistry of University of Buenos Aires (FOUBA). The primary objective was to evaluate the prevalence of EPL. The secondary objective was to identify potential risk indicators associated with their prevalence. **Methods**: Patients referred for first time to the Periodontics Department of FOUBA during April to June 2025 were consecutively selected. Clinical and radiographic examination was carried out. Categorical outcomes were described using proportions. The crude association between the prevalence of EPL and each of the recorded factors was determined by means of the chi-square test and a logistic regression analysis. **Results**: A total of 182 participants (128 women and 54 men) with a mean age of 50.8 (standard deviation = 15.6) years were included. The prevalence of participants with EPL was 14.8%. The average was 1.7 teeth per participant with a minimum of 1 tooth and a maximum of 5 teeth. In total, 85.2% of participants with EPL had stage III–IV generalized periodontitis, grade B or C. The logistic regression analysis identified periodontitis stage III–IV (OR = 5.9; 95% Confidence interval [1.9: 18.9] (*p* = 0.003)) as a potential risk indicator for EPL. **Conclusions**: The prevalence of participants with EPL in a teaching hospital of the University of Buenos Aires was 14.8%. EPL was more frequently found in participants with periodontitis stage III–IV. Periodontitis stage III–IV was considered a potential risk indicator of EPL.

## 1. Introduction

In 2018, the European Federation of Periodontology (EFP) and the American Academy of Periodontology (AAP) published a new classification system for periodontal and peri-implant diseases and conditions [1].

An endo-periodontal lesion (EPL) was defined as a pathological communication between the endodontic and periodontal tissues of a given tooth and classified as EPL with or without root damage according to the presence of signs and symptoms that affect their prognosis and treatment [2,3].

When traumatic or iatrogenic factors that could have caused root damage (fractures and perforations) are present, the most common manifestation of these acute form of EPL is an abscess accompanied by pain. EPL must be diagnosed as an EPL with root damage and its prognosis will be poor. Otherwise, chronic forms caused by the migration of microorganisms and inflammatory mediators between root and periodontium are characterized by the presence of deep periodontal pockets reaching to the apex and a negative response to pulp vitality test. In these cases, a periodontal evaluation of the entire mouth will be carried out, and lesions without root damage will be subclassified as EPL in patients without periodontitis or EPL in patients with periodontitis. Their prognosis will range from favorable to hopeless and their treatment will depend on the extent of the destruction of the periodontal tissues surrounding the tooth [3].

Although identification of the etiology of EPL is essential for proper treatment planning, it is not always possible to determine the primary cause. Therefore, epidemiological data become crucial to understand the prevalence, distribution, and associated factors of these lesions [4].

A study published in 2022 radiographically evaluated the prevalence of EPL according to this classification in a group of 866 participants treated at a teaching hospital in Germany. The prevalence was 4.9% and more frequent in participants with a diagnosis of stage III/IV periodontitis, concluding that prevalence might be higher in countries with greater prevalence of periodontitis [5].

Prior to this classification two studies were carried out according to 1999 International Workshop definitions and diagnostic criteria [6]. A cross-sectional study that analyzed the clinical records of patients treated at the Western University, California, USA concluded that 1 of 25 patients had rare forms of periodontal disease, combined periodontal–endodontic lesions being the most common (2.6%). While the other study found EPL in 14.89% of an Indian known population with caries. Concluding that a significant proportion of patients is affected by EPL and general dentists should be ready to recognize and manage rare periodontal diseases [7,8].

Sălceanu et al. conducted a retrospective study to identify independent risk factors and predictors for EPL in endodontically treated teeth with periapical pathology. Their findings highlighted age (≥60 years) and probing pocket depth ≥ 4 mm as significant independent risk factors. Other factors, such as marginal bone loss and occlusal considerations, appeared to play indirect roles. That study emphasized the necessity of early periodontal assessment in endodontically treated teeth and called for future research to explore the interplay between periodontal, endodontic, and systemic factors to improve predictive models [9].

As the evidence regarding the etiology, diagnosis, prognosis and treatment of EPL according to 2018 Classification of Periodontal and Peri-implant Diseases and Conditions (EFP/AAP) remains limited, a cross-sectional study was carried out at the Faculty of Dentistry of University of Buenos Aires (FOUBA). The primary objective was to evaluate the prevalence of EPL in individuals referred to the Periodontal Department of FOUBA. The secondary objective was to identify potential risk indicators associated with their prevalence.

## 2. Materials and Methods

### 2.1. Study Design

The present observational cross-sectional study is reported according to the Strengthening the Reporting of Observational studies in Epidemiology (STROBE) guidelines. It was conducted in accordance with the Helsinki declaration of human studies, and the research protocol was approved by the ethics committee of FOUBA (002/2025 CETICA-FOUBA). All participants provided their informed consent prior to the inclusion in the study.

### 2.2. Study Population

Patients referred for the first time to the Periodontics Department of FOUBA between April to June 2025 were consecutively selected based on the following inclusion and exclusion criteria.

#### 2.2.1. Inclusion Criteria

Patients over 18 years old who accepted to participate in the study and sign the informed consent.

#### 2.2.2. Exclusion Criteria

– Patients who attend for emergency treatment.
– Patients without X-rays.

### 2.3. Sample Size

The sample size calculation was carried out taking as reference the prevalence of 4.5% published by Ruetters M. in 2022 [5]. Considering that 2000 patients are treated at the periodontics department of FOUBA per year, a total of 181 participants were needed.

### 2.4. Data Collection

The primary outcome was the presence of EPL according to the classification of periodontal and peri-implant diseases of 2018 [2,3].

Furthermore, the following secondary variables were recorded by two trained and calibrated operators (CSH, BJ):

- Demographic data: Sex, age and nationality.
- Self-reported medical history (systemic status): Diabetes mellitus, cardiovascular diseases, oncological treatment, autoimmune diseases, medication intake (such as calcium fixatives) and smoking habit were recorded. Smoking participants were categorized according to the number of cigarettes they smoke per day in <5, 5 to 10, >10 per day and former smokers. Diabetic participants were categorized in pre-diabetes, type I and II.
- Periodontal diagnosis: The periodontal diagnosis of each participant was recorded according to the classification of periodontal and peri-implant diseases of 2018 [2,3]. For this purpose, a clinical and radiographic examination was performed.

The following clinical indices were recorded: probing depth (PD), recession (REC), clinical attachment loss (CAL), suppuration and bleeding on probing (BOP) [10] at 6 sites in all teeth with a North Carolina probe (UNC-15, Hu-Friedy, Chicago, IL, USA) at a pressure of 0.3 N. Furcation lesions according to the horizontal classification of Hamp by a Nabers probe [11] and degree of tooth mobility using the Miller scale [12] were recorded.

Radiographic evaluation was carried out by a panoramic radiograph and in the case of participants diagnosed with periodontitis, a series of radiographs was requested to complete the diagnosis and continue with the periodontal treatment.

In cases of teeth with a presumptive diagnosis of EPL pulp response was evaluated by a pulp sensitivity test to cold performed with a dichlorodifluoromethane cooling spray (Endo Ice, Klepp, Buenos Aires, Argentina^®^) and the response of the periradicular tissues by percussion and palpation was also recorded.

Bone loss and presence of intrabony defects (number of walls—angulation) were evaluated according to Goldman and Tsitoura [13,14] through a periapical radiograph taken by a RVG 5200 Radiovisiograph (Carestream Dental, Atlanta, GA, USA). Additionally, the presence of restauration, endodontic treatment, intra-root anchorage, caries, history of dental trauma and orthodontics was registered dichotomously.

Finally, the prognosis of the teeth was carried out according to the McGuire and Nunn’s classification [15,16].

### 2.5. Statistical Analysis

Participants and tooth level analyses were performed. EPL, demographic data, systemic status data (diabetes mellitus, cardiovascular diseases, oncological treatment, autoimmune diseases, medication intake and tobacco) and dental history (dental trauma, orthodontic treatment and periodontal treatment) were analyzed at the participant level. All other variables were assessed by tooth level analysis.

For quantitative outcomes, normality was assessed using the Kolmogorov–Smirnov test and box-plots, and the results were presented as the mean and standard deviation (SD). Categorical outcomes were described using proportions and contingency tables with the application of a Fisher’s exact or chi-square test. The crude association between the prevalence of EPL and each of the recorded factors was determined by means of a Student’s *t*-test and chi-square test.

In order to determine the potential risk indicators of EPL, individuals were classified based on the presence/absence of EPL (independently of their number). A logistic regression analysis was performed to identify factors (participant-related factors) associated with the presence of endo-periodontal lesions. Variables such as diabetes mellitus and periodontal diagnosis were recategorized as binary variables, diabetes mellitus as presence [pre-diabetes, type I and II]/absence, and periodontal diagnosis as health, gingivitis, and stage I–II vs. stage III–IV. The results were expressed as odds ratios (OR) with 95% confidence intervals (CIs).

The level of statistical significance was set at *p* < 0.05. All statistical analyses were performed with IBM SPSS Statistics version 29.0.1.0 software (IBM Corporation, Armonk, NY, USA).

## 3. Results

### 3.1. Sample Description

A total of 182 participants (128 women and 54 men) with a mean age of 50.8 (SD = 15.6) years were included in this study. Around 77.5% were Argentinian and the remaining 23.1% came mainly from neighboring countries (Paraguay, Bolivia and Peru); 25.8% were smokers, 12.6% had cardiovascular diseases and 7.1% (13 subjects) had diabetes mellitus (Table 1).

According to the classification of periodontal and peri-implant conditions of 2018 [2], 1.1% of the participants were diagnosed with periodontal health, 17.6% with gingivitis and the remaining 81.3% with different stages and grades of periodontitis, the most frequent stages being II (23.6%) and III (29.1%). Of the 182 participants, 4 acute periodontal lesions (3 periodontal abscesses and 1 necrotizing periodontal disease) were diagnosed. Overall, 27 participants (14.8%) had at least one tooth diagnosed with EPL.

### 3.2. Characteristics of Participants with Endo-Periodontal Lesions

A total of 27 participants were diagnosed with EPL according to the 2018 classification. The mean age was 58.6 (SD = 10.2), with an age range of 38–73 years. The average number of EPLs was 1.8 (SD = 1.2) teeth per participant with a minimum of 1 tooth and a maximum of 5 teeth. The distribution of the number of EPLs per participant showed that 55% of participants presented 1 lesion, 25.9% 2 lesions, and 18.5% ≥ 3 lesions. Within participants with EPL, 85.2% had stage III–IV generalized periodontitis, grade B or C, and 22.2% had diabetes mellitus (Table 2).

### 3.3. Characteristics of Teeth with Endo-Periodontal Lesions

**Figure 1:**
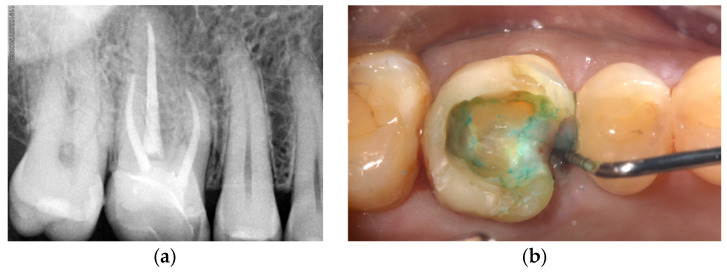
(**a**) Periapical radiograph of a maxillary molar with a vertical fracture, a two-wall intrabony defect, endodontic treatment and a plastic restoration. (**b**) Fracture line and localized increased in probing depth.

**Figure 2:**
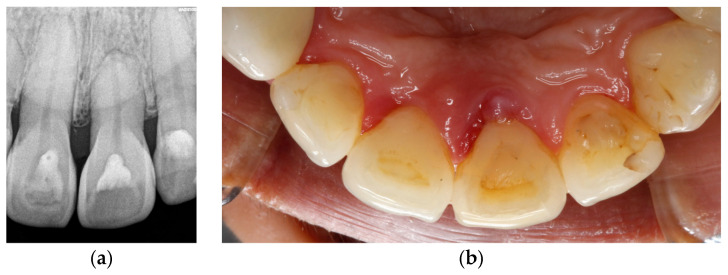
(**a**) Periapical radiograph of maxillary central incisor (2.1) showing root resorption. (**b**) Palatal restoration, edema, and traumatic occlusion.

**Figure 3:**
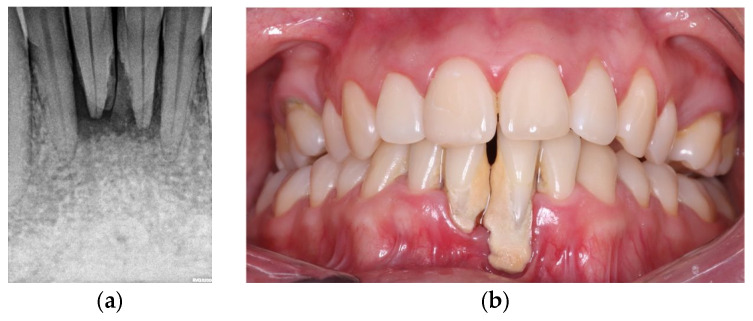
(**a**) Periapical radiograph of lower incisors (3.2–4.1) showing periapical process and one-wall intrabony defect. (**b**) Localized clinical attachment loss associated with labial piercing.

**Figure 4:**
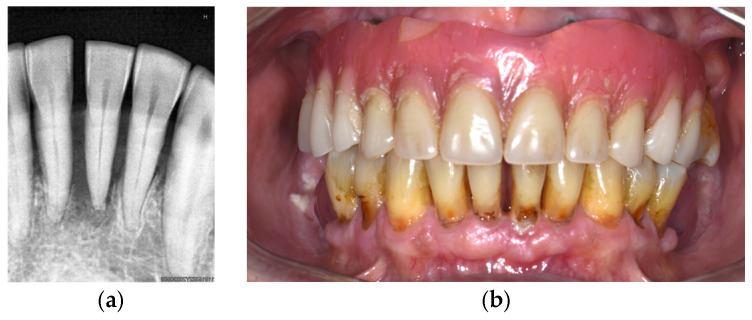
(**a**) Periapical radiograph of a lower incisor (3.1) with periapical process and one-wall intrabody defect. (**b**) Generalized periodontitis stage IV. Localized suppuration at tooth 3.1.

A total of 49 teeth (27 participants) were diagnosed with EPL according to the 2018 classification. Overall, 3 of them were diagnosed as EPL with root damage (1 premolar and 1 upper molar (Figure 1a,b) with vertical fractures and 1 upper incisor with a history of trauma with external root resorption (Figure 2a,b)) and 46 without root damage. In total, 2 lower incisors of a participant who had a history of trauma were subclassified as EPL in participants without periodontitis (Figure 3a,b) and the remaining 44 as EPL in participants with periodontitis (Figure 4a,b) (Table 3).

Clinical characteristics of EPL teeth are presented in Table 4. A mean PD of 7.8 mm (SD = 2.6), CAL of 10.3 mm (SD = 2.5) and REC of 2.5 mm (SD = 2.4) were observed. BOP was present at 100% of teeth and suppuration in 36.7% of them. The most frequently affected tooth was the lower incisor (22.4%), followed by upper molar (20.4%) and upper incisor (18.4%). Caries was found in 10.2% of teeth, 16.3% had some type of restoration, 4.1% had endodontic treatment (with interradicular anchorage), while the remaining 65.3% had no type of previous dental treatment. Sixteen teeth were multi-rooted, and twelve of them had furcation involvement (24.5%). Most teeth exhibited an intrabony defect (95.9%), mainly defects of the one-wall (42.9%) and two-wall (40.8%) types.

### 3.4. Individual Risk Indicators in Participants with Endo-Periodontal Lesion

General (sex, age, smoking habit, pre-existing systemic pathologies and medication) and periodontal-related outcomes (periodontal diagnosis) in individuals with EPL are described in Table 5. Statistically significant associations were observed between EPL and periodontal diagnosis (*p* = 0.013), diabetes mellitus (*p* = 0.025) and age (*p* = 0.005).

This association was further explored by a logistic regression model (Table 6). The adjusted logistic regression analysis (Model 4) identified periodontitis stage III–IV (OR = 5.9 CI 95% [1.9; 18.9]; *p* = 0.003) as a potential risk indicator of EPL.

## 4. Discussion

The present cross-sectional study was designed to evaluate the prevalence of EPL and identify potential risk indicators of EPL at a teaching hospital of the University of Buenos Aires and provide epidemiological information to the 2018 classification system for periodontal and peri-implant diseases and conditions.

A total of 182 participants (128 women and 54 men) with a mean age of 50.8 (SD = 15.6) years referred for the first time to the FOUBA periodontics department were included. Overall, 27 individuals (14.8%) had at least one tooth diagnosed with EPL, with an average of 1.8 (SD = 1.2) tooth per participant, with a minimum of one tooth and a maximum of five teeth. The prevalence of participants with EPL was 14.8% and the tooth most frequently affected was the lower incisor (22.4%).

Among participants with EPL, 85.2% had stage III–IV generalized periodontitis, grade B or C. These findings match with results published by Ruetters where most patients with EPL showed periodontitis stage III–IV, consistent with a higher maximal percentage of bone loss and age-adjusted bone-loss [5].

In this study, the mean age of participants with EPL (58.6) was higher than that of participant without EPL (49.4). Although a statistically significant association with age was observed (*p* = 0.005), it was not identified as a potential risk indicator, differing from a retrospective study conducted by Sălceanu et al. that identified age (>60 years old) and PPD > 4 mm as independent risk factors and predictors for EPL in endodontically treated teeth with periapical pathology and emphasized the necessity of early periodontal assessment in endodontically treated teeth and future research to explore the interplay between periodontal, endodontic, and systemic factors to improve predictive models [9].

In our study, the association between EPL and general factors (sex, age, smoking habit, pre-existing systemic pathologies and medication) was evaluated and a statistically significant association was observed in individuals with EPL and diabetes mellitus (*p* = 0.025). Despite the fact hyperglycemia, caused by this metabolic disorder, is a risk factor for the development and severity of periodontitis as well as alterations in pulp integrity, the adjusted logistic regression analysis did not identify it as a potential risk indicator of EPL [17,18]. These results differ from a study carried out by a tertiary care dental institution where the correlation between EPL according to Simons’ classification and systemic diseases was evaluated. The logistic regression analysis revealed that systemic diseases, especially diabetes mellitus (OR = 2.87), hypertension (OR = 2.45) and altered kidney function (OR = 2.13), were significantly associated with of endo-perio lesion severity and the group aged 56–75 years had the highest number of EPLs. Another study published by Rhee according to the 1999 classification system evaluated diabetes mellitus and tobacco, but no correlation with EPL was performed. The prevalence of EPL in Rhee et al. was 2.6%, but the authors concluded that general dentists should be ready to recognize and manage EPL [8,19,20].

The most common signs and symptoms of EPL reported by the 2018 classification of periodontal and peri-implant diseases and conditions (EFP/AAP) were deep periodontal pockets reaching or close to the apex and a negative or altered pulp vitality test. In accordance with this, the tooth-level analysis of the present study records a mean PD of 7.8 mm (SD = 2.6), CAL of 10.3 mm (SD = 2.5) and REC of 2.5 mm (SD = 2.4). BOP was present at 100% of teeth and suppuration in 36.7% of them [3].

In our study, the most frequently affected tooth was the lower incisor (22.4%), followed by the upper molar (20.4%) and upper incisor (18.4%). These findings differ from previous research utilizing this classification, which identified mandibular molars as the most affected teeth. We attribute this discrepancy to their exclusion of anterior teeth—often due to poor visualization in panoramic radiographs—as well as the exclusion of teeth with suspected root damage [5].

In this study, 65.3% of teeth had no type of previous dental treatment, 16.3% had some type of restoration, and 4.1% had endodontic treatment (with interradicular anchorage), while caries were found in 10.2% of teeth. A study carried out by Altaf evaluated the prevalence of EPL in an Indian population with caries and reported a prevalence of 14.9%, concluding that a significant proportion of participants was affected. A pilot study carried out at a Brazilian University Center evaluated the prevalence of EPL in 104 teeth referred for endodontic treatment, in most cases with pain (63.3%), caries/restorations (93.3%) and the absence of pulp sensitivity (56.7%). The molar teeth demonstrated a higher presence of probing depth (PD) ≥ 7 mm (38.3%) and higher PD mean (6.17 mm) than non-molar teeth (*p* < 0.05). True combined endodontic–periodontal lesion occurred significantly in molar teeth compared to non-molar teeth (*p* < 0.05). One-third of the sample had periodontal involvement, which demonstrates the importance of periodontal examination together with general clinical examination. In accordance with this study, we report sixteen multi-rooted teeth—twelve of them with furcation involvement (24.5%) [7,21].

Most teeth presented intrabony defects (95.9%), primarily of the one-wall (42.9%) and two-wall (40.8%) types. The extent of periodontal destruction compromised their prognosis, which was classified as hopeless in 59.1% of cases. Although no studies were identified regarding the management of an endo-periodontal lesion (EPL) associated with root damage, retrospective data have shown that combined endodontic and periodontal regenerative therapy significantly improves prognosis, retaining 92% of hopeless teeth when root damage is absent. Consequently, a recent systematic review emphasized the need for further research to evaluate treatment strategies for teeth with root damage and to determine the optimal sequencing of interventions. Furthermore, standardizing diagnostic criteria—based on the 2018 classification [1] (Caton et al., 2018)—and outcome reporting is essential to ensure comparability across future studies [22,23,24,25].

Since ELP is a rapid-onset clinical condition that severely compromises the prognosis of the tooth and its prevalence and severity seem to be related to age and systemic individual diseases, we consider that prospective studies are necessary to establish standardized treatment protocols for existing endo-periodontal lesions and to identify risk factors for their prevention.

Within the limitations of the study, it is important to acknowledge that sample size calculation was based on the quantity of patients referred to the periodontics department of a Latin American dental school. Additionally, systemic status was self-reported. Therefore, reference bias could be present, limiting the generalizability of these findings.

## 5. Conclusions

Despite the limitations of this study, it can be concluded that the prevalence of participants with EPL in a teaching hospital of the University of Buenos Aires was 14.8%. EPL was more frequently found in participants with periodontitis stage III–IV, which should be considered as a potential risk indicator of EPL.

## Figures and Tables

**Table 1:** Participant demographics, systemic health information and periodontal diagnosis.

**Age** (years) (mean [SD])	50.8 (15.6)
**Sex**	
Male (n [%])	54 (29.7)
Female (n [%])	128 (70.3)
**Nationality** (n [%])	
Argentine	141 (77.5)
Paraguayan	10 (5.5)
Bolivian	11 (6.0)
Venezuelan	3 (1.6)
Uruguayan	3 (1.6)
Peruvian	12 (6.6)
Colombian	1 (0.5)
Russian	1 (0.5)
**Pre-existing systemic pathologies and medication** (n [%])	
Cardiovascular disease	23 (12.6)
Hypertension	18 (9.9)
Other *	5 (2.7)
Diabetes mellitus	13 (7.1)
Type I	1 (0.5)
Type II	10 (5.5)
Pre-diabetes	2 (1.1)
Calcium fixators	6 (3.3)
Oncology treatment	6 (3.3)
Autoimmune disease	6 (3.3)
Rheumatoid arthritis	2 (1.1)
Other **	4 (2.2)
**Smoking habit (cig/day) (n [%])**	
Non-smoker	13 (73.6)
<5 cig/day	12 (6.6)
5–10 cig/day	18 (9.9)
>10 cig/day	12 (6.6)
Former smoker	6 (3.3)
**Periodontal diagnosis (n [%])**	
Health	2 (1.1)
Gingivitis	32 (17.6)
Periodontitis stage I	19 (10.4)
Periodontitis stage II	43 (23.6)
Periodontitis stage III	53 (29.1)
Periodontitis stage IV	33 (18.1)
**Participants with endo-periodontal lesion (n [%])**	**27 (14.8)**
**Distribution of the number of EPL per participants (n [%])**	
1 lesion	15 (55.6)
2 lesions	7 (25.9)
3 lesions	2 (7.4)
4 lesions	1 (3.7)
5 lesions	2 (7.4)

* Arrhythmia, heart failure, thrombosis; **: pemphigoid, scleroderma, thrombocytopenic purpura, Hashimoto’s thyroiditis. Cig: cigarettes; n: number of participants; EPL: endo-periodontal lesion; SD: standard deviation.

**Table 2:** Demographics, systemic health information and periodontal diagnosis of patients with endo-periodontal lesions.

**Age** (years) (mean [SD])	58.6 (10.2)
**Sex**	
Male (n [%])	10 (37.0)
Female (n [%])	17 (63.0)
**Nationality (n [%])**	
Argentine	25 (92.6)
Uruguayan	1 (3.7)
Colombian	1 (3.7)
**Pre-existing systemic pathologies and medication (n [%])**	
Cardiovascular disease	6 (22.2)
Hypertension	5 (18.5)
Other *	1 (3.7)
Diabetes mellitus	6 (22.2)
Type I	0 (0.0)
Type II	5 (18.5)
Pre-diabetes	1 (3.7)
Calcium fixators	0 (0.0)
Oncology treatment	1 (3.7)
Autoimmune disease	2 (7.4)
Rheumatoid arthritis	0 (0.0)
Other **	2(7.4)
**Smoking habit (cig/day) (n [%])**	
Non-smoker	18 (66.7)
<5 cig/day	2 (7.4)
5–10 cig/day	4 (14.8)
>10 cig/day	3 (11.1)
Former smoker	0 (0.0)
**Periodontal diagnosis (n [%])**	
Health	0 (0.0)
Gingivitis	1 (3.7)
Periodontitis stage I	0 (0.0)
Periodontitis stage II	3 (11.1)
Periodontitis stage III	15 (55.6)
Periodontitis stage IV	8 (29.6)
**Distribution of the number of EPL per participants (n [%])**	
1 lesion	15 (55.6)
2 lesions	7 (25.9)
3 lesions	2 (7.4)
4 lesions	1 (3.7)
5 lesions	2 (7.4)

*: Heart failure, **: scleroderma, thrombocytopenic purpura. Cig: cigarettes; n: number of participants; EPL: endo-periodontal lesion; SD: standard deviation.

**Table 3:** Distribution and frequency of teeth with endo-periodontal lesions according to the 2018 classification.

Endo-Periodontal Lesion (n [%])			
With root damage	Root fracture or cracking		2 (4.1)
	Root canal or pulp chamber perforation		0 (0)
	External root resorption		1 (2.0)
Without root damage	Periodontitis patients	Grade 1	6 (13.3)
		Grade 2	7 (14.3)
		Grade 3	31 (63.3)
	Non-periodontitis patients	Grade 1	0 (0)
		Grade 2	2 (4.1)
		Grade 3	0 (0)

n: number of teeth.

**Table 4:** Clinical and radiograph characteristics of teeth with endo-periodontal lesions.

**PD (mm)** (mean [SD])	7.8 (2.6)
**CAL (mm)** (mean [SD])	10.3 (2.5)
**REC (mm)** (mean [SD])	2.5 (2.4)
**BOP** (n [%])	49 (100)
**Suppuration** (n [%])	18 (36.7)
**Periodontal abscess** (n [%])	4 (8.9)
**Mobility** (n [%])	
**None**	10 (20.4)
Grade 1	2 (4.1)
Grade 2	19 (38.8)
Grade 3	18 (36.7)
**Tooth type** (n [%])	
Upper incisor	9 (18.5)
Lower incisor	11 (22.4)
Upper canine	2 (4.1)
Lower canine	2 (4.1)
Upper premolar	8 (16.3)
Lower premolar	1 (2.0)
Upper molar	10 (20.4)
Lower molar	6 (12.2)
**Furcation (n [%])**	
**No**	37 (75.5)
**Grade I**	1 (2.0)
**Grade II**	5 (10.2)
**Grade III**	6 (12.3)
**Dental history (n [%])**	
No treatment	32 (65.3)
Caries	5 (10.2)
Filtered restoration	8 (16.3)
Interradicular anchorage	2 (4.1)
Endodontic treatment	2 (4.1)
**Intrabony defects (n [%])**	
**None**	2 (4.1)
1 wall	21 (42.9)
2 wall	20 (40.8)
3 wall	6 (12.2)
**Angulation defect (n [%])**	
Wide	27 (57.4)
Narrow	20 (42.5)
**Prognosis (n [%])**	
Regular	2 (4.1)
Poor	9 (18.4)
Questionable	9 (18.4)
Hopeless	29 (59.1)

BOP: bleeding on probing; CAL: clinical attachment loss; n: number of teeth; PD: probing depth; REC: recession.

**Table 5:** Association of participants with endo-periodontal lesions with general and periodontal-related outcomes.

	Participants Without EPL (n = 155)	Participants With EPL (n = 27)	*p* Value
**Age** (mean [SD])	49.4 (16.0)	58.6 (10.3)	0.005
**Sex** (n [%])			0.364
Male	44 (28.4)	10 (37)	
Female	111 (71.6)	17 (63)	
**Smoking habit** (n [%])			0.447
Non-smoker	116 (74.8)	18 (66.7)	
<5 cigarettes per day	10 (6.5)	2 (7.4)	
5–10 cigarettes per day	14 (9)	4 (14.8)	
>10 cigarettes per day	9 (5.8)	3 (11.1)	
Former smoker	6 (3.9)	0 (0)	
**Cardiovascular disease** (n [%])			0.297
No	138 (89)	21 (77.8)	
Hypertension	13 (8.4)	5 (18.5)	
Other	4 (2.6)	1 (3.7)	
**Diabetes mellitus** (n [%])			0.025
No	148 (95.5)	21 (77.8)	
Type I	1 (0.6)	0 (0)	
Type II	5 (3.2)	5 (18.5)	
Pre-diabetes	1 (0.6)	1 (3.7)	
**Autoimmune disease (n [%])**			0.180
No	151 (97.4)	25 (92.6)	
Rheumatoid arthritis	2 (1.3)	0 (0)	
Other	2 (1.3)	2 (7.4)	
**Oncology treatment (n [%])**			0.624
Yes	5 (3.2)	1 (96.3)	
No	150 (96.8)	26 (3.7)	
**Calcium fixators (n [%])**			0.725
Yes	2 (1.3)	0 (0)	
No	153 (98.7)	27 (100)	
**Periodontal diagnosis (n [%])**			<0.001
Health	2 (1.3)	0 (0)	
Gingivitis	31 (20.1)	1 (3.7)	
Periodontitis stage I	18 (11.7)	0 (0)	
Periodontitis stage II	40 (26.0)	3 (11.1)	
Periodontitis stage III	38 (24.7)	15 (55.6)	
Periodontitis stage IV	25 (16.2)	8 (29.6)	

EPL: endo-periodontal lesions.

**Table 6:** Logistic regression models for the analysis of endo-periodontal lesions.

	MODEL	Parameter	OR	95% CI		*p*-Value
				Lower Bound	Upper Bound	
**Crude association**	**MODEL 1** *p*-value ≤ 0.001 Adjusted R2 = 0.076	Constant	0.174			<0.001
		**Diabetes mellitus**	6.041	1.852	19.705	0.003
	**MODEL 2** *p*-value ≤ 0.001 Adjusted R2 = 0.179	Constant	0.044			<0.001
		**Periodontal diagnosis**	8.306	2.739	25.185	<0.001
	**MODEL 3** *p*-value 0.005 Adjusted R2 = 0.075	Constant	0.023			
		**Age**	1.039	1.010	1.068	0.008
**Adjusted model**	**MODEL 4** *p*-value = <0.001 Adjusted R2 = 0.224	Constant	0.017			<0.001
		**Diabetes mellitus**	3.243	0.905	11.618	0.071
		**Periodontal diagnosis**	5.925	1.861	18.864	0.003
		**Age (years)**	1.019	0.987	1.052	0.246

## Data Availability

Data generated during the current study are available from the corresponding author upon reasonable request.

## Abbreviations

The following abbreviations are used in this manuscript:

EPL	Endo-periodontal lesion
EFP	European Federation of Periodontology
AAP	American Academy of Periodontology
FOUBA	Faculty of Dentistry of University of Buenos Aires
STROBE	Strengthening the Reporting of Observational studies in Epidemiology guidelines
PD	Probing depth recession
CAL	Clinical attachment loss
REC	Recession
BBOP	Bleeding on probing
SD	Standard deviation
